# Strengthening health technology assessment for cancer treatments in Europe by integrating causal inference and target trial emulation

**DOI:** 10.1016/j.lanepe.2025.101294

**Published:** 2025-04-09

**Authors:** Heiner C. Bucher, Frédérique Chammartin

**Affiliations:** Division of Clinical Epidemiology, Department of Clinical Research, University Hospital and University of Basel, Basel, Switzerland

**Keywords:** Health technology assessment, Causal inference, Estimand, Target trial emulation, Europe

## Abstract

Health Technology Assessment (HTA) for reimbursement of all new cancer drugs in the European Union (EU) will be evaluated for all member states by a central European HTA starting in 2025. EU HTA guidelines for applicants under these new regulations put the focus on meta-analysis of aggregated randomized trial data and are in contrast with the growing number of cancer drug approvals by drug regulators that are based on single arm studies and the needs in the rapidly evolving field of oncological drug development. We advocate to broaden the methodological approaches for HTA by including observational data based causal inference methodology and target trial emulation into the assessments of comparative effectiveness. Causal inference estimates causal estimands, effect measures that reflect a population level effect in terms of contrasts of counterfactual outcomes in the same patients and are directly measured in the target population by modeling of hypothetical intervention. Target trial emulation allows with the use of causal inference to estimate causal effects by mimicking pragmatic trials that evolve apart from randomization like a trial. We illustrate the potential of causal inference for HTA and provide an introduction into causal inference methodology for health scientists involved in HTA.

**Search strategy and selection criteria.**

References for this Personal View were identified through searches of PubMed with the search terms (causal inference) AND (Methodology [MeSH Major Topic]) from 2005 until March 2025 and by the study of groundbreaking books and publications on causal inference published by Pearl J, Rubin DB, Greenland S, Hernan M. and Robins J (See references for details). Only papers published in English were reviewed. The final reference list was generated on the basis of their relevance to the broad scope of this Personal View.

## Introduction

Health technology assessments (HTA) is a multidisciplinary process to determine with the use of state of the art methods the value of a health technology for decision making in health care.^1^ A central concept of HTA is the evaluation of the additional benefit of a new technology compared to a standard treatment in the target population under real world conditions. In theory, the use of real-world data for the assessment of comparative effectiveness, a key dimension of HTA for reimbursement decisions, would be the logical consequence. However, the quality of real-world data and the risk of bias from the use of observational data are of major concern for choosing this approach.

## European union health technology assessment methodological guidelines for joint clinical assessments

The recently released EU HTA guidelines on direct and indirect comparison that provide the methodological background for the Joint Clinical Assessment put for these reasons a strong focus on evidence synthesis from meta-analysis of direct and indirect comparisons of randomized trials as the main methodology for comparative effectiveness documentation.^2^^,^^3^ These guidelines will be applied in 2025 for all new cancer drugs and will provide orientation for the Joint Clinical Assessment under the new EU HTA Regulations with the goal to centrally harmonize and standardize HTAs among all member states.

The guideline’s directions, however, do not well reflect the advances towards individualized cancer drug developments and the approval policy of cancer drugs by regulators. Between 2015 and 2020 the Food and Drug Administration (FDA) approved 43% and the European Medicines Agency (EMA) 21% of drugs for non-orphan diseases based on single arm studies with no control arm.^4^ Meanwhile survey data of HTA decision policies from Germany, France and UK show that applications and approvals for reimbursement of cancer drugs that were based on external controls were entirely rejected,^5^ or highly divergent among HTA bodies.^6^^,^^7^ It is therefore critical for thousands of patients in need of access to new treatments in the EU that the harmonization process for HTA provides a methodological framework that allows for more solid and coherent assessment of evidence for the effectiveness of cancer drugs that are based on indirect comparison.^8^

We argue that currently accepted methods in HTA should be critically reevaluated and methodological approaches for HTA broadened by integrating causal inference methodology and target trial emulation into the assessments of comparative effectiveness. This would also allow for synergies for approval and reimbursement processes. We present basic principles of causal inference for applicants, assessors, and decision makers of HTA and illustrate its potential for HTA.

### Estimands, an underused concept in HTA

The scoping process for HTA requires the formulation of a PICO question (Population, Intervention, Comparator, Outcome) with the aim to translate the health policy decision makers’ question into a research question. The PICO approach does not obviously align with the regulatory approval process for single arm studies with no controls or for approvals based on randomized trials using estimands. The concept of estimands was proposed by the International Council for Harmonisation (ICH) and adopted by FDA for drug approval guidance for the industry but has not yet penetrated into HTA.^9^, ^10^, ^11^, ^12^ An estimand is defined by the following attributes: treatment, target population, outcome of interest, *population level summary estimate*, and the strategy for (post-randomization) intercurrent events.^13^ The last two attributes are not sufficiently specified within the PICO framework. An estimand is a precise definition of a treatment effect from a research question that applies to a clearly defined population. Estimands have a crucial role in causal inference because a causal effect is conceived in terms of contrasts of counterfactual outcomes *in the same patients* that are directly measured in the target population by modeling of hypothetical interventions.

There are several arguments why causal inference should be attractive to HTA, particularly in the absence of randomized evidence. The need for a definition of estimands sharpens the understanding for study protocol development and observational or interventional data generation processes that may help to align needs of regulators and HTA bodies. Counterfactual based analysis has an interventional approach towards modeling the likelihood of events under changing conditions in the target population and is deemed to estimate effects of more complex treatment algorithms that are typical in cancer treatment and thus more informative for health policy decision makers. Outside the scope of this paper, counterfactual based analysis is also able to handle intercurrent events in randomized studies through a hypothetical scenario of non-occurrence.

### Lack of causal interpretation of relative effect measures from meta-analysis

The methodological focus of HTA on meta-analysis relates to the systematic approach to evidence identification and synthesis with the derivation of relative effect measures with protection from confounding if only properly randomized trials are included. A random effect meta-analysis model, for example, is often considered as the top of the pyramid of evidence, notably due to its way to explicitly handle between study heterogeneity. EU HTA guidelines consider meta-analysis of randomized trials – by nature an observational method due to its ex-post approach – generally as less biased than analyses from observational cohort studies that do not control for unmeasured confounding. There are many arguments to challenge this view.^14^^,^^15^ Meta-analysis of aggregated data assumes that treatment effects from different randomized studies are drawn from the same underlying distribution, an assumption that is referred to as *exchangeability*.^16^ While exchangeability holds for each single randomized trial included in a meta-analysis this is not necessary the case for the interpretation of any pooled estimate from meta-analysis of aggregated data. In practice, effect modifiers can introduce variability between studies and challenge exchangeability. Exchangeability in meta-analysis implies similarity in regard to effect modifiers across treatment comparison and is not verifiable. It can only be indirectly addressed by exploring the heterogeneity of summary estimates.^17^ Network meta-analysis adds an additional component of heterogeneity that can result from divergent estimates from trials of direct versus indirect comparisons.^16^ The usual low number of trials in meta-analytic data sets or trial networks, which is particularly the case in HTA applications of cancer drugs, makes a trustworthy exploration of heterogeneity impossible.^16^ Also, meta-analysis of aggregated data is limited in the analysis of treatment related effect modification, impacting the ability of conducting relevant subgroup analyses. In the therapeutic world of oncology heterogeneity due to tumor biology, treatment response and resistance, however, is a reality and must be addressed by adequate methods to account for effect modification.^18^

Meta-analysis further assumes to estimate common effects in the available studies that are derived from a random sample from the trial superpopulation (fixed effect model) or from a sample from the distribution of study effects (random effect model).^19^^,^^20^ Both assumptions hint to a common problem of meta-analysis, the difficulty to define the target population and a population-level effect measure, two key elements of the estimand definition. Approaches for a causal framework for meta-analysis have been developed which all rely on random sampling assumptions of trials from a superpopulation that may not hold or make the interpretation of causal effects difficult to interpret.^19^^,^^21^ These methods have not yet been applied in HTA. Thus, meta-analysis produces pooled estimates that do not allow for a causal interpretation of treatment contrasts for the target population of interest and are therefore of questionable relevance for health policy decision makers.^20^^,^^22^

## Basics of causal inference

Causal inference aims to identify causal relationships and quantify effects of an intervention under *changing conditions* from observational data.^23^^,^^24^ The approach requires a strict separation of concepts of association and causation.^25^ Causal inference allows the definition of a causal question, comparing a new treatment with a standard treatment, as done in HTA. We are interested in the causal contrasts and consequences of *what would have happened if* patients were given or not a particular treatment X = x. This is typically done with Rubin’s potential outcomes or counterfactual outcomes framework^26^ (Box 1) where the Average Causal Effect (ACE) of an exposure on outcome is defined as the difference in expected outcomes under two levels of treatment such as ACE = E [Y^x = 1^ − Y^x = 0^], where Y is the outcome of interest and X takes value 0 when untreated and 1 when treated. Hence, Y (1) and Y (0) are *potential outcomes* and refer to outcomes that would be observed under different levels of exposure. This is in opposition to factual outcome that refers to experienced observed outcomes based on real data and counterfactual outcome that refers to hypothetical outcomes that would have been observed under a different scenario. If the two potential outcomes differ, we say that treatment X has a causal effect on the outcome Y. An example illustrating the counterfactual concept is given in Box 1.

Box 1 **World of counterfactual knowledge–example.**

**Table 1:** Potential outcome framework.

Patient	Type	Outcomes under different treatment strategies	
		Untreated	Treated
1	Doomed	Dead	Dead
2	Helped	Dead	Alive
3	Harmed	Alive	Dead
4	Immune	Alive	Alive

Let’s consider a cohort of N patients (n = 1, …, 4) belonging to one of the four possible types doomed, helped, harmed or immune, a dichotomous treatment variable X ∈{0,1}, where 0 = untreated and 1 = treated, and an outcome Y∈{0,1}, where 0 = alive and 1 = dead. Let’s Y (0) and Y (1) be the outcomes under the treatment value X = 0 and X = 1, respectively. For example, patient n = 1 of the type “doomed” would die if he/she does not receive the treatment or if he/she would receive the treatment, whereas patient n = 4 of the type “immune” would survive when treated and untreated. A causal effect is defined when Y (1) ≠ Y (0) which is the case for patients of types helped and harmed but not for patients of types doomed and immune. For obvious reasons, only the factual outcome can be observed (i.e. the outcome under the strategy that was given to a patient) and the counterfactual is hypothetical (see Table 2). For example, if the treatment assigned to a patient takes value 1 (X = 1; treated), we observe the factual outcome Y (X = 1) and Y (X = 0) is counterfactual. Y (1) and Y (0) are named *potential outcomes* and refer to outcomes a patient would experience under a given treatment strategy (Table 1). If the two potential outcomes differ, we can say under certain assumptions (specified in the text) that treatment X has had a causal effect on Y. Similarly, we can define an average causal effect in the population in probabilistic terms such as P(Y(1) = 1) − P(Y(0) = 1) or as a difference in expected outcomes E [Y (1)] − E [Y (0)]. If the two outcomes do not differ, i.e. Y (1) = Y (0), the average causal effect in the population is null and the common statement used in causal inference through the *null hypothesis of no causal average effect* is true.

For simplicity, we illustrated the world of counterfactual with the simplistic outcome dead/alive. Causal inference methods can model binary, continuous as well as time to event outcomes. Outcomes like overall or progression-free survival and response rates are however more realistic study outcomes in oncology.

**Table 2:** Factual and counterfactual outcomes.

Patient	Treatment	Outcome	Type	Observed outcome	
				Y (X = 0)	Y (X = 1)
1	Treated	Dead	Doomed	?	Dead
	Untreated	Dead		Dead	?
2	Treated	Alive	Helped	?	Alive
	Untreated	Dead		Dead	?
3	Treated	Dead	Harmed	?	Dead
	Untreated	Dead		Dead	?
4	Treated	Alive	Immune	?	Alive
	Untreated	Alive		Alive	?

Counterfactual outcomes are not observed and depicted with a question mark.

To better understand the features and role of counterfactuals in causal inference for observational data analysis we turn for a moment to the randomized controlled trial. In a randomized trial the Average Treatment Effect (ATE) is defined as the difference between the expected outcomes for patients randomized to intervention and to control: ATE = E [Y (1)] − E [Y (0)]. Because of randomization, the assignation of X is known and fixed by the investigator (X∈{0,1}). Proper randomization and trial conduct ensure that the treatment allocation is independent from patient characteristics Z and that treatment X causes Y. Therefore, the potential outcomes are independent from the treatment assignation X which is expressed as (Y (0),Y (1)) ⫫ X for all values x, and patients in the exposed arm (X = 1) and in the unexposed arm (X = 0) have the same distribution of outcomes that would be observed in a hypothetical situation where everyone is exposed and unexposed to X. Potential outcomes Y (0) and Y (1) under randomization are independent from treatment assignment, the exquisite feature of the experimental design.^27^ This situation is named *exchangeability*, a key assumption of causal inference. Consequently, association in a trial is causation.

**Fig. 1:**
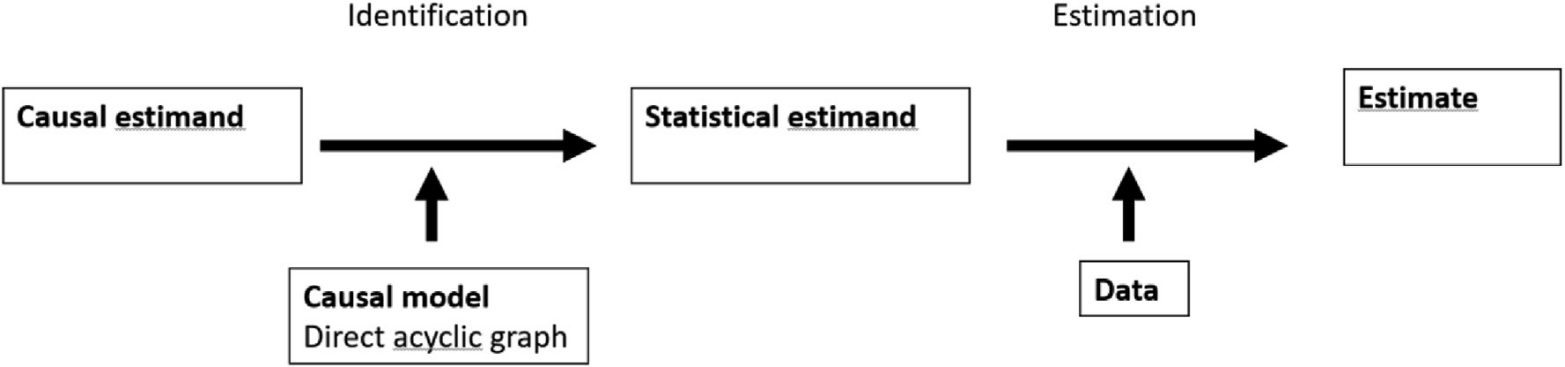
Process flow chart in causal inference.

Now consider an observational study where the treatment X is administrated in a way that is associated to some patient characteristics, besides any causal link from X to Y, a condition we refer to as confounding. In such situation, treated and untreated groups are not comparable, and the key assumption of exchangeability is violated. To claim a causal effect, strong statistical adjustments with causal inference methods are needed, with the basic assumption that there is no unmeasured confounding. This strong assumption cannot be tested. These methods require a good understanding of the causal mechanisms and a careful assessment of the underlying assumptions namely that exchangeability conditional on the measured covariates holds and allows to identify the causal effect of interest. As depicted in Fig. 1, there are three key processual elements to causal inference^28^: specification of the causal estimand, identification and estimation.

### Specification of causal estimand

The first step in causal inference is to clearly define the quantity that we aim to estimate, namely the causal estimand. The definition of the causal estimand is done within the potential outcome framework through the specification of key components which include population, treatment, outcome, potential outcome, and causal contrast of interest. For example, a causal contrast of interest can be the difference in potential outcomes under different treatment strategies. It is also important to define whether the causal estimand is intended to describe an effect *in the entire population* which is called a *marginal effect* (*average treatment effect*) or effects among subpopulations which are called *conditional effects* where one conditions on specific characteristics of the population (e.g. patients in less *vs* more advanced disease stages). This has consequences for the analytical approach and model choice.

### Identification

**Fig. 2:**
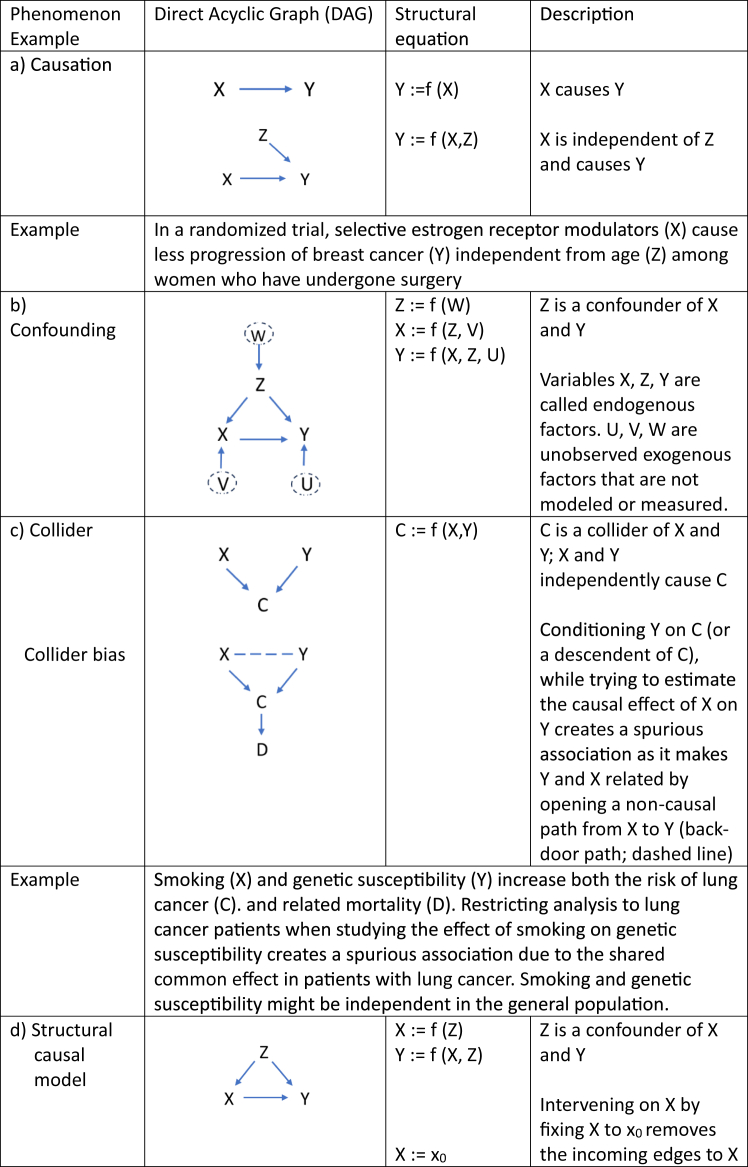

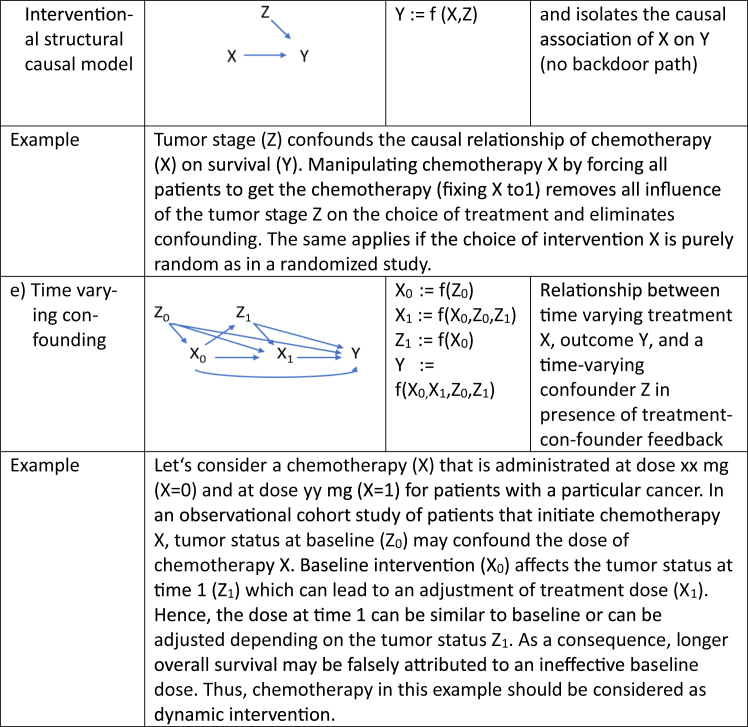
Different types of structural causal models and related functions.

The key question of causal inference analyses is whether we can formulate the causal estimand as a *statistical estimand*, a quantifiable measure that can be estimated from the observed data given a statistical model. In other words, how can we estimate the controlled (post-intervention) distribution from data governed by the pre-intervention distribution?^23^^,^^29^ To achieve this goal, assumptions are required, and we need to show that these assumptions hold with the data of interest. A causal effect is *identifiable* if it can be uniquely estimated from the pre-interventional observed data and when the necessary assumptions about the data and the underlying causal relationships are not violated.^23^ This entails *exchangeability*, *positivity* and *consistency*. These assumptions cannot be tested statistically but rather must be justified based on theory e.g. on disease mechanisms and existing evidence about the causal question under study. This requires expert knowledge and explicit and transparent statements about causal mechanisms and involved factors that must allow for an external check of model assumptions by the critical reader. In an observational study the pre-interventional distribution does not fully reflect the true causal effect of an intervention due to the presence of confounders. It must be inferred from the observed data via structural equations where we model data in a particular way. With the use of causal diagrams the structure of confounding, the bias introduced due to common causes of treatment, and outcome must be detailed. In Fig. 2b the confounder Z is a common cause of treatment X and outcome Y. The links between the confounder Z and treatment X and confounder Z and outcome Y introduces a backdoor path that leads to biased estimates of the casual effect of X on Y. Causal modeling is about blocking this backdoor path by controlling for confounder Z and achieve conditional exchangeability. As a caveat, estimates from the modeling approach will be biased if the necessary confounding variables are not, or imperfectly measured, or mis-specified. Modeling details are given in Box 2.

Box 2 **Identification process in causal inference.**

The central question, called *identification*, in causal inference is whether the causal estimand P (Y ǀ do(X = x)) can be estimated from the statistical estimand that refers to the joint distribution of observed data P (X,Y,Z). Of note, we use here the “do”^23^^,^^29^ to express the hypothetical probability P(Y) if treatment X is set to x in the population. Identification involves a causal model with structural equations that can be exemplified with directed acyclic graphs (DAGs).

**Direct acyclic graph (DAG)**

DAGs comprise nodes representing random variables and arrows. An arrow pointing from one variable to another indicates a direct causal effect. The lack of an arrow means that there is no causal effect between two variables. We take the simplest example and design a DAG to assess the causal effect of treatment X on outcome Y in the presence of Z, a variable representing the severity of disease as an example (see Fig. 2b).

The *Markov assumption* ensures that each variable in the DAG is conditionally independent of all other variables, with the exception of its parents or descendants. A DAG is truly acyclic, meaning that there are no arrows flowing back from any nodes. In addition, all noise terms U, V, and W (so-called exogenous factors) that are not measured are represented with dashed circles and are jointly independent, meaning that they are not dependent on each other.

**Specification of a structural causal model (SCM)**

Each function of a SCM is a formal representation of a causal process described in the DAG and defines how each variable is influenced by its direct causes. Absence of a variable on the right-hand side of an equation means that the variable has no direct effect on the left-hand side variable. In an interventional structural causal model, we set the intervention X to a fixed value and consequently cut the dependence of X on its causes. It is then easy to isolate the causal association of X on Y (see interventional SCM in Fig. 2d). To mimic what would happen if we intervened on X in observational data, where a set of confounders Z affect both X and Y, we have to introduce the *backdoor adjustment*, which stipulates that conditioning on Z blocks the confounding path and allows for an unbiased estimate of the causal effect if Z satisfies the backdoor criterion. So, if we find an appropriate set of covariate Z that are not descendants of X and that blocks all non-causal paths between X and Y that include an arrow pointing into X, the causal effect of X on Y after setting X = x can be estimated using the backdoor adjustment formula:

$$P\;\left(Y|\text{do}\;\left(X=x\right)\right)=\sum_{z}P\left(Y|X=x,Z=z\right)P\left(Z=z\right)\text{.}$$

Thus, we have a link between a causal estimand expressed as a potential outcome (left-hand side of the above formula), and a statistical estimand (right-hand side of the above formula) that is expressed as conditional probabilities. Adjusting for confounding ensures *conditional exchangeability* for a fair comparison between the different levels of X and a valid causal inference. Assumptions of *no unmeasured confounding* and *correct model specification* are essential to satisfy the exchangeability assumption and ensure an unbiased estimate of the causal effect.

### Estimation

Estimation is central to determine the causal effect of interest from observed data, using the statistical estimand introduced above. Different estimation methods such as propensity score matching, the inverse probability weighting and G-computation formula are available and discussed below. For assumptions, advantages and disadvantages of models other sources should be sought.^27^

- *Propensity score* matching is the most used tool to address confounding in observational studies and estimate causal effect in HTA and one of the methods listed in the EU guidelines.^3^ Propensity score measures the probability of treatment in individuals given a set of covariates L such as P [X = 1|L].^28^ Individuals with an estimated probability (or propensity score) close to 0 have a low probability of receiving a treatment and those close to 1 have a high probability of treatment. Propensity scores are used to match individuals from treated and untreated groups to compare outcomes between the matched groups. Propensity scores are a one-dimensional summary score of covariates of multiple dimensions and have several disadvantages.^30^ Individuals with the same score can have quite different distributions of covariates and the inclusion of all ‘thinkable’ covariates that are not true confounders may lead to imprecise or inflated variance estimates (variance-bias trade-off). Lack of overlap in scores between treated and untreated patients may lead to matching or stratification difficulties and selection bias by restricting the analysis to individuals with ranges of scores that best overlap. Too loose criteria for matching can lead to the potential violation of exchangeability and compromise the validity and reliability of the results. Too tight criteria can substantially reduce sample size and limit generalizability of the findings. One main limitation of propensity score matching, however, relates to the impossibility to model complex dynamic treatment algorithms and to address time varying confounding. Main advantages include the simplicity of the method and a possible direct comparison of treated and control groups if desirable.
- *Inverse probability weighting* (IPW) is the second technique listed in the EU guidelines. IPW creates a pseudo-population by weighting individuals by the inverse (reciprocal) of the conditional probability of their treatment exposure history at each visit (propensity score), given the value of previous confounders. In this pseudo cohort the measured confounders are balanced to overcome selection bias. An unbiased causal effect can be derived, given that there is no unmeasured confounding. IPW techniques can also additionally encompass stabilized and censoring weights.
- The *G computation formula* is a standardization method that uses parametric regression models. It provides a general framework to estimate causal effect that involves parametric modeling and does not necessarily rely on a specific backdoor path and structural models (see Box 3 for the identification process with respect to the G computation formula). The technique provides a broader method for the estimation of probability distributions of time varying covariates and offers the flexibility to naturally address time-varying confounders and treatment-confounder feed-back such as depicted in Fig. 2e. This cannot be addressed by conventional statistical approaches, which hold confounders at a fixed level. Direct Acyclic Graphs (DAGs explained in Box 2) help to illustrate this problem: identification requires to maintain exchangeability by blocking all paths between exposure and outcome. A typical situation in clinical medicine arises if intermediate variables like for example prognostic markers that are measured when initiating treatment and during follow-up affect future treatment decisions. Typical examples are drug dosing (treatment intensification or reduction due to toxicity or drug–drug interaction), change in risk factor exposure (e.g. blood pressure, alteration in tumor receptor status), or emerging drug resistance due to genetic mutations or insufficient adherence. Causal estimates are computed by averaging the confounder specific mean outcomes under the exposure of interest (e.g., treat always, never treat, treat if prognostic markers is above or below a cut-off for treatment initiation) over the entire follow-up period in a hypothetical cohort. The method is also the method of choice to address dynamic interventions due to its ability to handle complex temporal and causal relationships. Hence, the G computation formula is not restricted to a single intervention. It is also applicable to simultaneous or sequential interventions that allow to analyze time varying treatment with time varying confounding.^23^ Other typical time varying variables are the so-called colliders that are influenced by both the exposure and the outcome (Fig. 2c). Controlling and conditioning on a collider in the causal pathway between the exposure and the outcome leads to collider bias by establishing a spurious association between exposure and outcome. It is therefore extremely important to recognize colliders to avoid such biases and produce accurate causal estimates.
  Box 3 **Identification with respect to the G computation formula.**
  In a simple causal DAG such as the one depicted in Fig. 2a where X is the parent of Y, and Z has no parent, we assume that each variable is conditionally independent of its non-descendants given its parents. In other words, any variable on the DAG is independent of any other variables for which it is not the cause. This is the so-called causal Markov assumption, which implies that the joint distribution P (X, Y, Z) can be factorized as:
  P(X,Y,Z)=P(Y|X,Z)P(X|Z)P(Z)
  Let’s now intervene on the intervention X by setting the value for that node constant and let all other nodes unchanged. The relationship between X and its parents is cut and the joint distribution becomes truncated. The above factorization can therefore be simplified as:
  P(Y,Z|do(x))=P(Y|X=x,Z)P(Z|X=x)
  Finally, we can marginalize out (‘average out’) the confounder Z by summing up all values of Z with the generalized intervention formula also known as the ‘G computation formula’ to get the probability to observe a specific outcome Y given that an intervention X is given:
  $${P\;\left(Y\;|\;\text{do}\left(x\right)\right)=\sum }_{Z}P\left(Z\right)P\left(Y\;|\;Z,X=x\right)$$
  Hence, the G computation formula provides a broader method that involves conditional modeling and that does not necessarily rely on specific backdoor paths.
- *G-estimation* models link the exposure variable of interest with the counterfactual outcome under no exposure during follow-up to the weighted sum of time spent in a given exposure status.^30^ The method estimates parameters of structural nested models. The predicted exposure at each data measuring point (visit) based on the history of previous exposure and covariates and counterfactual outcomes is then estimated by logistic regression. In an iterative modeling process the causal value of the exposure variable is then estimated based on pre-treatment distribution of variables in the data set, in order to render exposure independent of the counterfactual outcomes given previous treatment and confounder history. These adjustments are done at each visit over the entire follow-up period and achieve control of time dependent confounding.

There are three so called generalized methods (G-methods) that can address time varying confounding: *Inverse probability weighting, G-computation formula and g-estimation*.^27^

Accounting of time varying confounding and treatment confounder feedback is one of the key features and advantages of the G computation formula which allows for the modeling of more complex treatment algorithms, and better reflect the clinical practice of oncology where treatments are modified due to drug toxicity or disease progression. Effect measures have *a direct causal interpretation* as outcome differences under different treatment values of X in the population of interest, given that all key assumptions (exchangeability, positivity and consistency) are met. This is one of the most important features of causal inference that should alert HTA bodies and panels to give causal inference methodology more attention.

### Target trial emulation

Let us consider first a pragmatic randomized trial where treatment is openly assigned with active treatment comparisons (no placebo), and where participants are monitored according to clinical routine. In an *intention to treat analysis* a causal effect is estimated as causal contrast in individuals assigned to treatment with randomization at baseline that are followed-up until censoring or end of follow-up.^27^^,^^31^ As participants may deviate from the protocol for various reasons like drug-toxicity, disease progression, non-adherence or switch to the alternate trial regimen or other drugs, the *per-protocol effect* is of interest which is defined as the contrast of the outcome of continuous treatment exposures between initiation at randomization until the end of follow-up. This may be the even more relevant contrast which involves a comparison of dynamic and realistic strategies in a real world setting that is important for decision making.

If the conduct of a pragmatic randomized trial is not possible a *target trial* based on observational data may be emulated.^32^ This involves defining in a protocol the inclusion criteria, start and end of follow-up, treatment strategies, outcomes of interest, causal contrasts, and data analysis plan. Definition of a causal effect from an observational data-based target trial requires to be explicit about the compared strategies that contrast well-defined counterfactuals. In mimicking a RCT as close as possible, causal contrasts of a treatment are not defined like in a trial by randomized assignment but according to the initiation of each treatment strategy.

A crucial point of target trial emulation is the definition of baseline (time zero) when eligibility is assessed and met, treatments get initiated, and follow-up starts. A typical problem arises if individuals in a cohort fulfill inclusion criteria for initiating a treatment strategy at several timepoints, which can introduce bias because of selection affected by treatment.^33^ If data collection is fixed (e.g. every 6 months) or patient specific (e.g. from medical records) a new target trial can be modeled at each time point or prespecified selected timepoints.^34^ Another approach is forming two clones of each individual and allocate each clone to one of the treatment strategies. Clones are censored when they are no longer consistent with the initial treatment strategy.

In clinical routine a treatment decision can for clinical, administrative, and other reasons often not be immediately started when criteria to initiate the treatment are met (e.g. waiting time for drug resistance test data). G-methods allows also for the definition of a grace period after time 0 (e.g. 3 months) during which initiation of a treatment is still considered acceptable. During the grace period an individual’s observed data is then consistent with more than one treatment strategy. Cloning, censoring, and inverse probability weighting at each time point due to adjustment of time dependent confounding require adequate adjustment with the use of g estimation for target trial emulations.

With high cohort data quality (well characterized cohort with full set of all known covariates at baseline, regular and maximally complete follow-up for time varying covariates, and adjudicated outcome assessments) the target trial framework provides the methodological ground for the causal analysis of observational studies. Apart from randomization there are no fundamental differences in the analysis of a per protocol effect in a randomized trial in real world settings with prolonged follow-up and protocol deviations and the analysis of an observational data based emulated target trial. In both studies there is a need for adjustment by g-estimation for time varying prognostic factors that are associated with drop out (selection bias) and treatment (confounding).

Although randomization is the standard for comparative effectiveness, the developments in cancer drug research and regulatory decision-making show growing numbers of drugs not being evaluated by randomized trials. Randomized trials also have many problems as a gold standard in practice and may suffer from many problems of internal validity and limited transportability of results to the real-life setting. Therefore, causal inference and target trial emulation should become an option for comparative effectiveness research if excellent observational data is available. Ideally, this requires prospective data collection with continuous quality control and regular sufficiently narrow follow-up examinations. Uptake of the methodology to address important oncological treatment questions is increasing,^35^, ^36^, ^37^, ^38^, ^39^ with publications using proposed methods for time dependent confounding adjustment.^40^, ^41^, ^42^, ^43^ Reporting guidelines for target trial emulation are under way that may facilitate the use of the methodology.^44^

## Conclusions

Every methodology has its limitation and is not necessary or appropriate for every application.^25^ The methodological preference of current EU HTA guidelines for evidence synthesis from meta-analysis of randomized trials is problematic and in disharmony with the growing number of approvals of single arm cancer drugs by regulators.

In the absence of evidence from randomized controlled trials causal inference offers the possibility to coordinate the data generation process for newly developed cancer drugs that will allow for a timely definition of estimands and modeling of causal contrasts of data from single arm approval studies and external controls. Target trial emulation and g-estimation are valid tools to model causal contrast of treatments by avoiding or minimizing well-knowns bias in observational study protocol development and conduct. A formal causal framework provides a valuable tool for prospective planning of observational studies or pragmatic trials in a real world setting that may cover the entire drug development phases II to IV studies. The framework is based on transparent specification of all necessary assumptions inherently needed for causal inference model development and data analysis and allows for a critical interpretation of results. Creation of a real-world database infrastructure of high quality and granularity is an absolute priority to advance HTA that is based on both evidence from studies with external controls and pragmatic randomized controlled trials.^45^

## Contributors

HCB was responsible for conceptualisation, search of literature, reviewing the methodology and published educational material for causal inference, writing of the first and final draft and review and editing of the manuscript. FC was responsible for literature review, writing of all parts on reviewed statistical methods of the manuscript, drafting and editing of figures and text boxes, and final review and editing.

## Declaration of interests

The corresponding author received no funding for writing of the manuscript. Frédérique Chammartin is supported by grants from the Moritz Strauss Foundation and the Swiss National Science Foundation.
